# Trials and tribulations: cross-learning from the practices of epidemiologists and economists in the evaluation of public health interventions

**DOI:** 10.1093/heapol/czy028

**Published:** 2018-03-27

**Authors:** Timothy Powell-Jackson, Calum Davey, Edoardo Masset, Shari Krishnaratne, Richard Hayes, Kara Hanson, James R Hargreaves

**Affiliations:** 1 London School of Hygiene and Tropical Medicine (LSHTM), Faculty of Public Health and Policy, 15-17 Tavistock Place, London WC1H 9SH, UK; 2 London International Development Centre (LIDC), 36 Gordon Square, London, WC1E 0PD, UK; 3 London School of Hygiene and Tropical Medicine (LSHTM), MRC Tropical Epidemiology Group, Faculty of Epidemiology and Population Health, Keppel Street, London WC1E 7HT, UK

**Keywords:** Epidemiology, economics, evaluation, randomized controlled trial

## Abstract

The randomized controlled trial is commonly used by both epidemiologists and economists to test the effectiveness of public health interventions. Yet we have noticed differences in practice between the two disciplines. In this article, we propose that there are some underlying differences between the disciplines in the way trials are used, how they are conducted and how results from trials are reported and disseminated. We hypothesize that evidence-based public health could be strengthened by understanding these differences, harvesting best-practice across the disciplines and breaking down communication barriers between economists and epidemiologists who conduct trials of public health interventions.

Key Messages

- The randomized controlled trial is commonly used by both epidemiologists and economists to test the effectiveness of public health interventions.
- There is convergence between disciplines in a number of areas, most notably in the appreciation for protocols, pre-specifying outcomes and the analysis approaches and registering trials.
- Differences between disciplines suggests that more can be done to incorporate behavioural theory into trials, improve the reporting of trial results and share data.
- We hypothesize that evidence-based public health can be strengthened by understanding differences in how economists and epidemiologists conduct trials of public health interventions and harvesting best-practice across the disciplines.

## Introduction

The randomized controlled trial is widely used in biomedical and social science. Trials of public health interventions—as opposed to clinical trials (Collier 2009)—date back at least to the study of tuberculosis prophylaxis in the 1960s (Comstock *et al.* 1967). Prominent examples in economics of the early use of trials include the RAND health insurance experiment (Manning *et al.* 1987). But it is in the past two decades that there has been a surge of interest in trials among economists, especially those working in international development. This has led to debate about their usefulness (Deaton 2010). Such debates are also alive and well in public health (Bonell *et al.* 2012).

Epidemiologists and economists both use trials to test the impact of public health interventions. For example, both epidemiologists and economists have tested the impact of deworming on education outcomes (Awasthi *et al.* 2013; Miguel and Kremer 2004), and the effects of cash-transfers on HIV (Baird *et al.* 2012; Pettifor *et al.* 2016). However, we have noticed differences in practice between the two disciplines. By understanding better these differences and looking at best practices across the two disciplines, we hypothesize that evidence-based public health can be strengthened.

In this article, we propose that while there is both significant overlap between, and heterogeneity of practice within, the two disciplines, there are three underlying areas of difference in the way economists and epidemiologists undertake trials: the purpose for which trials are used, the conduct of trials and the way that the results of trials are reported and disseminated. We describe these differences, illustrating with examples and end by suggesting some areas where best practice could be transported in either direction across the disciplinary divide, and call for ongoing efforts to support inter-disciplinary communication and collaboration. The focus of the paper is on public health interventions that typically have a behavioural element to them; we are less interested in clinical drugs trials where the intervention works primarily through a biological mechanism and can be tested using double-blind placebo-controlled trials. In an article of this nature, we recognize that it is difficult to avoid making generalizations. Heterogeneity within the disciplines probably dwarfs that between them. Moreover, we have not systematically reviewed the literature and think it likely that counter-examples to those we have raised in the paper can be identified. Nevertheless, we believe that our observations will resonate with readers and stimulate debate.

## How are trials used?

Trials offer a transparent approach to get an unbiased estimate of the causal effect of an intervention on an outcome, in a particular setting. They address the question: does it work? Both epidemiologists and economists use trials to evaluate whether an intervention works. Trials of this nature are sometimes referred to as ‘effectiveness trials’ (in epidemiology) or ‘policy evaluations’ (in economics). The intervention is delivered in conditions that are (argued to be) similar to real-world implementation of the intervention.

Public health interventions often aim to change human behaviour, be that of healthcare providers, patients or the public in general. However, since most public health interventions are social, the effects are likely to vary when tried with different populations in different places, and even at different times. We need to understand why an intervention worked, and for whom, if the findings are to be useful in other settings.

Both disciplines support the idea of replicating trials in different settings with a view to making findings more generalizable (Angrist and Pischke 2010; Cook and Campbell 1979). However, our observation is that beyond replication, economists and epidemiologists confront the challenge of addressing external validity in different ways, and this affects the reasons why, and how, they do trials in the first place. There is also the fact that economics is a social science—with a rich tradition of developing formal theory of behaviour in the mathematical expression of a set of ideas and principles—in a way that epidemiology is not.

Epidemiologists tend to design trials in multi-disciplinary teams, in part because there is no generally accepted model of human behaviour within epidemiology. They bring in experts to help develop interventions and, in doing so, draw on theory from a range of other disciplines, such as health promotion. Some evaluations are informed by a so-called theory of change (Breuer *et al.* 2016) that provides a conceptual map of how the intervention is intended to work, grounded in the context where the trial is taking place. To understand how an intervention worked, mechanisms of effect are explored through process evaluation and the use of qualitative research methods (Moore *et al.* 2015).

Economists appear to favour other strategies to help generalize findings. We observe that economists seem more willing to have multiple trial arms to test variations in the intervention along dimensions that can tell us something about how it works while epidemiologists tend to focus on simple trials to test the best designed and most policy relevant candidate intervention. For example, in an epidemiologist-led trial of conditional cash transfers for HIV prevention in rural South Africa, there were just two study arms: families in the intervention arm received $36 per month conditional on school attendance; families in the control arm received nothing (Pettifor *et al.* 2016). In contrast, in an economist-led trial of cash for HIV prevention in Malawi, almost no two individuals in the intervention arm received exactly the same amount of money, since amounts of cash that went to different recipients were varied randomly, as was the conditionality attached (Baird *et al.* 2012).

Some economists argue for greater use of ‘mechanism experiments’, where the intervention is not designed to be implementable in the real-world, but instead helps to test a behavioural theory (Ludwig *et al.* 2011). For example, a two-stage pricing design was used in a trial in Zambia to test several competing theories of why higher prices of health products increase actual use (Ashraf *et al.* 2010). Epidemiologists, it should be stressed, do use trials to test directly theories relevant to public health, but often these relate to a biological mechanism rather than behavioural theory. For example, the recent HPTN052 trial tested the theory that successful antiretroviral treatment of an HIV-infected individual would reduce their infectiousness, and that this in turn could reduce transmission to partners (Cohen *et al.* 2016). HIV-discordant couples were enrolled to the placebo-controlled study but the specific intervention with couples was intended as a ‘proof of principle’ to inform later trials of scalable approaches to preventing HIV transmission through the provision of ARVs to those infected with HIV. But on balance we suggest that economists appear more willing than epidemiologists to explicitly design trials that *test* aspects of human behaviour.

As a follow on, economists sometimes use ‘structural models’ to incorporate or interpret the findings from a trial (Attanasio *et al.* 2012; Heckman 2010; Todd and Wolpin 2006). In essence, this approach develops a model of behaviour making explicit its assumptions, takes the experimental results, fits the model and makes predictions about the effect of changing the policy or the setting (Card *et al.* 2011; Ludwig *et al.* 2011). This approach to making predictions draws on the rich tradition in economics of developing formal theory and combining it with empirical research. There is a parallel in epidemiology where empirical findings are extrapolated from one setting to another using mathematical models of infectious disease transmission (White *et al.* 2008) that sometimes also incorporate aspects of human behaviour (Medley *et al.* 2015).

## How are trials conducted?

How a trial is undertaken and how the data are analysed affect the risk of drawing the wrong conclusions about the effects of the intervention. Both disciplines take this risk seriously, but we note that there appear to be differences in what are considered standard, or best, practices in each discipline.

It has long been standard in epidemiology to publish study protocols and register trials. Protocols describe the entire trial in detail: the hypothesis, rationale, study population, study design, primary and secondary outcomes, sample size calculations, data collection and analysis methods, and timeline for the study. Detailed statistical analysis plans are usually specified before a trial is complete and made available in the public domain. Trial registration—to limit publication bias—is now a requirement for most journals, and there are many registers, such as the ISRCTN Registry, Clinicaltrials.gov, the registry of the National Institutes of Health in the United States and the EU Clinical Trials registry. It should be noted that these practices have been strongly influenced by the developing practice of trials in medicine. While these procedural aspects of trials have historically been neglected by economists, recent years have seen convergence with the practice of epidemiologists (AEA 2016; Casey *et al.* 2012).

In epidemiology trials, for example, the study size is usually determined based on having sufficient power for a hypothesized effect on a single primary outcome (Smith *et al.* 2015). In contrast, power calculations have not traditionally been used by economists. However, as experimental approaches have gained in popularity, the statistical design of experiments has now become part of econometric textbooks. The use of power calculations to set the sample size is now common, even if these details are rarely reported in journal articles (Cameron and Trivedi 2005; Duflo *et al.* 2008). The evidence, beyond just trials, suggests that most empirical research in both economics and health is underpowered (Ioannidis *et al.* 2017; Turner *et al.* 2013).

Economists tend to analyse trials using ordinary least squares regression, regardless of whether the outcome is continuous (e.g. income) or dichotomous (e.g. being vaccinated). In epidemiology, trials are often analysed using simple statistics or regression methods designed for the specific type of outcome under study. Common analysis methods include logistic regression for binary outcomes, Poisson or Cox models for time-to-event data, or linear regression for continuous outcome data. In both disciplines, these modelling approaches make it straightforward to include the baseline characteristics of the units to improve precision and examine effects within different sub-groups of the population. Confidence intervals are near-universally reported by epidemiologists, while in economics the reporting of standard errors is the norm. It is worth noting that standard errors are not useful for ratio effect estimators—often used in epidemiology trials—because of highly skewed distributions.

Epidemiologists pre-specify a hierarchy of outcomes: a primary outcome and a number of secondary outcomes. Considerable emphasis and effort is given to objectively-measured clinical outcomes. Outcomes that were not pre-specified are analysed, but the results of such analyses are, at least in theory, treated with caution because of the potential for false positive findings to arise as a result of selective reporting. Economists appear to place less emphasis on such a hierarchy, in some cases specifying a large number of outcomes and then using statistical methods to deal with the problem of multiple hypothesis testing (Anderson 2008; Kling *et al.* 2007). This is attractive when interventions are multi-faceted, such as, for example, poverty reduction or agricultural projects that produce effects on many outcomes, none of which can be considered more important a priori. There is also recognition that such interventions can have unexpected and unintended effects which are important to capture.

Finally, economists appear more willing to give higher profile to analyses that go beyond the basic analysis of a trial. This might include, for example, using random assignment as an instrumental variable to estimate effects of treatment on the treated as opposed to intent-to-treat estimates, when there is partial compliance (Finkelstein *et al.* 2012). Other methods, such as quantile regression analysis and regression discontinuity designs, have been used in the analysis of trials to understand variation in who benefits from an intervention (Banerjee *et al.* 2015; Duflo *et al.* 2011). While such techniques are also used by statistical epidemiologists, the primary analysis of epidemiology trials of public health interventions appears to strongly privilege transparent, simple, pre-specified analysis of a limited number of outcomes.

## How are trial findings reported and disseminated?

Researchers in both fields want trials to inform policy and improve lives. However, the approaches of the two disciplines to reporting and dissemination appear different, perhaps reflecting the research-into-policy routes that have historically been common in each discipline.

Epidemiological public health gives primacy to evidence synthesis, through systematic reviews and meta-analysis, as established in clinical medicine. The World Health Organization plays a normative role in advising on best practice, and has an established approach to evidence appraisal based on systematic review. Systematic reviews combine all relevant evidence and quantify the quality of the evidence using methods intended to reduce the risk of bias or the role of reviewer subjectivity. In contrast, economics does not appear to have such a tradition or well-established architecture for doing systematic reviews. With one notable exception (Croke *et al*. 2016), reviews conducted by economists tend to be narrative in nature, include an appraisal of relevant theory, and are conducted by a leader in a field rather than according to an agreed method. The generalizability of the findings of a study is thus more likely to be argued within trial papers themselves.

To facilitate the research-into-reviews route, trialists in medicine and public health have developed the Consolidated Standards of Reporting Trials (CONSORT) statement that standardizes reporting, with a strong focus on showing the completeness of data and loss to follow up (Begg *et al.* 1996). While the guidelines are for reporting, they also shape the way that epidemiologists conduct trials by pre-specifying the information to be collected. A consequence of the strict template is an expectation for quick publication after data collection, since analysis decisions should have been made at the protocol stage. Economists are not formally guided by such standards which has meant that, historically at least, papers published in economics journals, while considerably longer, have not always provided sufficient information for the assessment of risk of bias (Boone *et al.* 2013). Economists do have a strong tradition of, and expectation towards, the sharing of data and analysis code that may in part compensate for the lack of stringent guidelines for reporting. It seems likely that epidemiologists will follow suit in this respect as medical journals increasingly require data to be made publicly available (Warren 2016), but at the current time the practices appear quite different.

A final difference we note is in the use of working papers. These are draft versions of papers that economists circulate online and at seminars to gather criticism, establish ‘property rights’ over an idea, and garner interest. The time between data collection and formal publication appears much longer in economics (Card and DellaVigna 2013) than in epidemiology. The practice of presenting working papers at conferences and seminars before publication allows for a thorough public review, in addition to the peer-review process required by journals. However, since working papers can be revised, misleading conclusions may be promulgated while the delay in producing a final version poses a challenge for contributing to policy in a timely manner.

## Way forward

The design of public health trials often brings perspectives from clinical medicine and the behavioural and social sciences. Key decisions about the purpose, design, conduct and reporting of trials often lie with either epidemiologists or, increasingly, economists. These scientists share a common appreciation of the benefits of trials. We think it likely that there is considerable heterogeneity in practices within each discipline. However, we propose that there are key areas in which the two disciplines differ and we have sought to pin point where these differences might lie. We acknowledge that counter-examples to the generalizations we have raised throughout this paper can be made and future research should systematically review the literature for more robust evidence of such differences and their implications. Nevertheless, we have time and again heard from our colleagues of instances where communication and understanding across the disciplines, even among those using similar research designs to address similar questions, is challenging. We wish to see greater collaboration across disciplines, and perceive that there have been instances of confusion and miscommunication that work against this. It is these that we seek to break down, and our paper is an attempt to identify factors at the root of this misunderstanding so that best practices from each discipline can be taken up.

Recent growth of the use of trials in economics is mostly a positive development. The fact that trials lower the risk of bias compared to alternative designs is why epidemiologists and economists have embraced them. We see convergence in the appreciation for protocols, pre-specifying outcomes and the analysis approaches that will be used, and registering protocols with a third party to reduce publication bias. Increased data-sharing is another element of the transparency agenda now supported by both disciplines. This convergence has come from best practices already being shared across disciplines.

Trials led by economists often seek to incorporate behavioural theory into the design of trials or in the interpretation of results. For example, trials of financial incentives that use multiple treatments to map the shape of the relationship between the size of an incentive and the outcome is motivated by theory that helps to generalize findings (Cohen and Dupas 2010). While such ‘dosing’ approaches are common in trials of medicines, we perceive they are less common in trials of public health interventions designed by epidemiologists. There is an increasing emphasis on theories of change and process evaluations alongside such trials to assess implementation, mechanisms of change and relevant contextual factors. However, there may be something for epidemiologists to learn from economists in how they use relevant theory to both inform evaluations of behavioural interventions and interpret their results.

Economics has a strong tradition based around working papers. Unlike in epidemiology and the medical sciences, papers can take several years to be published in the most reputable journals (Card and DellaVigna 2013). While there is much credit in the debate and discussion this system catalyses, we see a tension between this way of working and the desire for results from trials to rapidly make it into evidence synthesis processes and thereby inform policy. We suggest that the primary pre-specified analyses of all trials conducted by economists and epidemiologists alike should be reported in short, CONSORT-format products as soon as is feasible. This should not get in the way of the longer process of challenge and theoretical development that is current practice in economics.

Finally, we believe it important that synthesis for policy makers should use the whole evidence base in an unbiased way. This implies a need for understanding of different types of evidence within review teams, and a greater appreciation of similarities and differences between disciplines. It also implies we need to make work more visible across our disciplinary and publishing divide to support better evidence-based policy making in public health.

## Funding

Richard Hayes receives support from the MRC UK and DFID - MRC Grant Reference MR/K012126/1. This award is jointly funded by the UK Medical Research Council (MRC) and the UK Department for International Development (DFID) under the MRC/DFID Concordat agreement and is also part of the EDCTP2 programme supported by the European Union.

*Conflict of interest statement*. None declared.
